# Enhancing Honey Bee Health: Evaluating Pollen Substitute Diets in Field and Cage Experiments

**DOI:** 10.3390/insects15050361

**Published:** 2024-05-16

**Authors:** Hyunjee Kim, Olga Frunze, Jeong-Hyeon Lee, Hyung-Wook Kwon

**Affiliations:** 1 Convergence Research Center for Insect Vectors, Incheon National University, Incheon 22012, Republic of Korea; beamed79@hanmail.net (H.K.); frunzeon@gmail.com (O.F.); 2 Department of Life Sciences, College of Life Science and Bioengineering, Incheon National University, Incheon 22012, Republic of Korea; jeonghyeon@inu.ac.kr

**Keywords:** honey bee, pollen substitute diets, nutrition, health, development, amino acid contents

## Abstract

**Simple Summary:**

Globally, honey bee colonies face numerous threats, such as habitat loss, diseases, chemical exposure, and climate change, with malnutrition being a significant factor in colony decline. Beekeepers struggle to provide adequate pollen during seasonal pollen dearth when it is not readily available to honey bees, prompting the use of pollen substitute diets to support colonies. Our study compared a new pollen substitute diet with a control through field and cage experiments, finding that Diet 1 significantly improved honey bee health. This was evidenced by an increase in population, a greater brood area, higher consumption levels, preference, altered colony weight, honey production, honey bee weight (dried head and thorax), and *vitellogenin* expression levels. These improvements were correlated with the amino acid content of each diet, offering potential solutions to boost honey bee health and combat colony decline.

**Abstract:**

Honey bees (*Apis mellifera* L.) play vital roles as agricultural pollinators and honey producers. However, global colony losses are increasing due to multiple stressors, including malnutrition. Our study evaluated the effects of four pollen substitute diets (Diet 1, Diet 2, Diet 3, and Control) through field and cage experiments, analyzing 11 parameters and 21 amino acids. Notably, Diet 1 demonstrated significantly superior performance in the field experiment, including the number of honey bees, brood area, consumption, preference, colony weight, and honey production. In the cage experiment, Diet 1 also showed superior performance in dried head and thorax weight and *vitellogenin* (*vg*) gene expression levels. Canonical discriminant and principle component analyses highlighted Diet 1’s distinctiveness, with histidine, diet digestibility, consumption, *vg* gene expression levels, and isoleucine identified as key factors. Arginine showed significant correlations with a wide range of parameters, including the number of honey bees, brood area, and consumption, with Diet 1 exhibiting higher levels. Diet 1, containing apple juice, soytide, and *Chlorella* as additive components, outperformed the other diets, suggesting an enhanced formulation for pollen substitute diets. These findings hold promise for the development of more effective diets, potentially contributing to honey bee health.

## 1. Introduction

Honey bees (*Apis mellifera* L.) are vital agricultural pollinators cultivated for honey and other hive products [1,2]. Globally, high honey bee colony losses occur due to the effects of multiple stressors [3]. This decline in honey bee populations has had adverse impacts on both the economy and ecology, posing a significant threat to crop production and human well-being [4,5]. Several factors contribute to the decline, including habitat loss, diseases, pathogen infections, chemical exposures, and climatic changes [3,6,7]. Among these factors, malnutritional stress plays a significant role in colony decline [6]. The quality of a colony’s nutrition impacts its health, strength, caste differentiation process, and lifespan regulation, particularly for colonies preparing for overwintering or beginning population growth in early spring [8,9].

Honey bee nutrition primarily relies on the intake of two key resources collected by foragers from flowering plants: pollen and nectar. Pollen is converted to beebread, providing essential nutrients, such as proteins, lipids, vitamins, and minerals [10]. The concentration of crude protein and amino acid content in pollen varies depending on its origin, ranging from 4 to 40.8% and from 14.51 to 98.93 mM, respectively [11]. Young nurse workers consume beebread to satisfy their nutritional requirements and produce protein-rich secretions, such as royal jelly, from their hypopharyngeal glands, which are then used to nourish developing broods [12]. Nectar, on the other hand, is converted into honey and serves as the primary carbohydrate source for honey bees, with amino acids being the second most abundant compound [13]. Honey serves as the main source of energy for various bodily functions, including muscular activities and temperature regulation, and facilitates crucial processes in specific organs in honey bees [14].

It can be challenging for beekeepers to find sufficient and high-quality pollen resources for their colonies during seasonal pollen dearth when pollen is not readily available to honey bees [15]. Beekeepers often use pollen substitute diets to aid colonies through sparse periods or prepare them for spring buildup [15]. To develop a pollen substitute diet, soy flour and brewer’s yeast were utilized as protein supplements, serving as the foundational components of the diet [15]. Proteins have significant effects on physiological processes, such as immune response, parasite tolerance, survival, worker lifespan, and drone reproductive quality, as well as on brood rearing, adult population development, and royal jelly production [16]. They are typically composed of diverse amino acids bonded together through peptide linkages [17]. These constituents are vital for providing organisms with essential nitrogen and sulfur, which cannot be substituted by carbohydrates and lipids [17,18].

Many beekeepers commonly utilize commercial or artificial diets to supplement the apparent nutritional supply [8,14]. A pollen substitute diet should ensure a sufficient supply and balance of amino acids, which are important for maintaining health, vitality, and productivity in bee colonies [14,19]. However, certain pollen sources may lack essential amino acids that honey bees cannot produce internally but rely on to fulfill their nutritional requirements [2]. Studies have shown that a higher content of essential amino acids is superior to a lower content in terms of egg-laying time and egg number [20]. Insufficient or imbalanced amino acid intake can lead to developmental abnormalities, weakened immune function, reduced lifespan, and decreased colony productivity [21].

An effective pollen substitute diet should not only benefit the health of honey bees but also be palatable to them [15]. Bees and other insects, such as ants, butterflies, and flies, have been observed to show preferences for single amino acids or mixtures of them, contributing to the attractiveness of nectars and serving as critical components in the diets of flower visitors [22,23,24,25].

Environmental variability impacts pollen substitute diet efficacy, influenced by factors like geography, climate, weather, seasons, and pollen availability. Results from controlled lab settings may not reflect real-world conditions accurately, necessitating cautious interpretation. Consistency between lab and field studies is crucial for reliable outcomes. [15]. However, to the best of our knowledge, there have been no studies comparing the effects of pollen substitute diets through both field and cage experiments up to now.

In this study, we conducted experiments to elucidate the efficacy of pollen substitute diets across diverse environmental settings under both field and controlled cage conditions. Under field conditions, we examined the following parameters: the population density of honey bees, brood area expansion, consumption dynamics, dietary preference, colony mass dynamics, honey production yields, and hygienic behavior manifestations, as well as measurements of the dried head and thorax weight ratio and *vitellogenin* (*vg*) gene expression levels. Additionally, in cage conditions, we evaluated the consumption rate, *vg* gene expression levels, protein content in the head, and diet digestibility. Furthermore, we conducted a comparative analysis between the performance of honey bees fed the pollen substitute diets and the amino acid composition of these diets, providing valuable insights into their nutritional adequacy and suitability for honey bee health and development.

## 2. Materials and Methods

### 2.1. Preparation of Pollen Substitute Diet

Our developed pollen substitute diets included Diet 1, Diet 2, and Diet 3. The detailed components and concentrations of each diet are listed in Table 1. Diet 2 and Diet 3 were selected based on their positive colony performance in our previous study [26]. Diet 1 was a blend of ingredients aimed at enhancing the functions of Diet 2 and Diet 3. To improve the nutritional quality of the diets, we incorporated distinct functional additives: soytide (CJ Global Food and Bio Company, Seoul, Republic of Korea), apple juice (Jaan Company, Seoul, Republic of Korea), and *Chlorella sorokiniana* powder (Cheonil Herbal Medicine, Daegu, Republic of Korea). Apple juice was included in Diet 2 due to its association with extended longevity in adult codling moths, *Cydia pomonella* [27]. Soytide was incorporated into Diet 3 as a functional additive. It comprised fermented soybean flour, characterized by its high protein content and low antinutritional and allergenic properties. These properties facilitate development and nutrient utilization [21,28]. In Diet 1, apple juice, soytide, and *Chlorella* powder were added as functional supplements. This decision was based on our previous study, which showed the most significant honey bee colony growth in the diet supplemented with apple juice, followed by excellent colony growth in the diet supplemented with soytide [26]. Additionally, *Chlorella* powder was included because another research team demonstrated its beneficial effects on honey bee health [9].

### 2.2. Field Experiment Design

**Figure 1:**
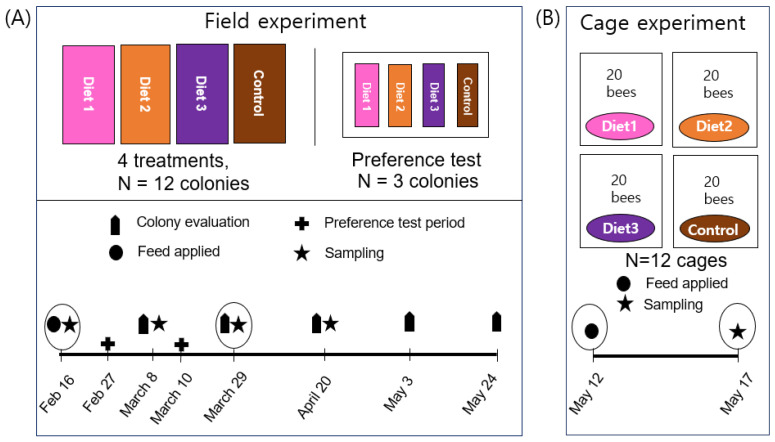
Schematic overview of experiment design. (**A**) Field experiment: Fifteen colonies were used for field experiments. (**B**) Cage experiment: Twelve cages were used for cage experiments. The diets were provided during the timeframe indicated by the empty egg-shaped circles.

The current study was conducted with fifteen honey bee colonies in Gangneung, South Korea, from 16 February to 24 May 2023. Each experiment consisted of three replications. There were three artificial diets (Diet 1, Diet 2, and Diet 3), and a commercial diet, Megabee (Castle Dome Solutions, Helena, AR, USA), which is a mixture of plant-based proteins with no pollen and is widely employed by beekeepers in the U.S., served as a control in this study [27]. Approximately 1200 g of diet was applied from 16 February to 29 March to evaluate the influence of the diets. Since nearby flowers began blooming on 29 March, no further diets were supplied thereafter. The specific field experiment design is described in Figure 1A.

### 2.3. Field Experiment

Honey bee population, capped brood area, consumption, preference, colony weight, honey production, hygienic behavior, and dried head and thorax weight were estimated in this study. The honey bee population within each colony was determined by visually estimating the percentage of adult bees present on each side of every comb in the colony [29]. These individual percentages were then combined to determine the total percentage representing the colony’s adult bee population. Subsequently, the population size was determined by multiplying this total percentage by the number of honey bees typically found on each fully occupied side of a Langstroth frame. Previous assessments established that each fully covered side of a frame contained approximately two thousand honey bees, providing the basis for this calculation [30].

The capped worker brood area was measured approximately every 21 days using the Puchta method [31,32]. The capped brood areas were calculated using the formula S = 3.14 × A/2 × a/2 (S: area; A: long axis of the ellipse; and a: short axis of the ellipse). We estimated the change in the capped brood area from the initial measurement within each colony. The consumption was measured as the difference between the fresh weight of the supplemental diet and the weight of the remaining diet (g per colony) (consumption = total weight of the diet given − total weight of the remaining diet). To assess preference, a 400 g portion of each diet was prepared, and all four diets were placed on the upper side of the frames within a single colony for 12 days. Preference is defined as a change in diet mass [28].

Colony weight was measured by carefully transferring the entire hive onto an electronic balance. Honey production was quantified by calculating the difference in colony weight between 3 May and 24 May, corresponding to the period of acacia flower blossoming near our apiary.

Hygienic behavior was measured by counting the number of cleaned cells among 49 pin-killed cells after 24 h.

To measure the weight of the dried head and thorax, three honey bees from each replicate field sample collected on 8 March were dissected over dry ice. The dissection distinguished between the head, thorax (excluding legs and wings), and abdomen. We separately combined the individuals in each group into groups of three heads and three thoraxes. The weights of the heads and thoraxes were determined by drying them in an oven at 60 °C overnight.

### 2.4. Cage Experiment

Three capped brood frames were selected from three robust honey bee colonies at the apiary of Incheon National University in May 2023. These frames were then transferred to an incubator until the newly hatched bees emerged. Upon emergence, the newly hatched honey bees within 24 h were randomly placed, with 20 honey bees per wooden cage (70 mm × 90 mm × 100 mm) (Figure 1B). The wood cages included a plastic door that could slide open and down. Additionally, a 15 mL falcon tube with a hole was placed in the upper part of each cage and filled with boiled water for sterilization. Each diet was presented in a 5 mL plastic dish to allow ad libitum access to food.

The cage experiment was conducted for five days at a controlled temperature of 33 ± 2 °C and relative humidity of 55 ± 5% within the incubator. The dead honey bees in the cages were taken out and recorded every day.

To evaluate diet consumption, the remaining food was weighed, and the initial amount supplied was subtracted from this value. We considered evaporation and substracted the evaporation amount from the remaining diet. After five days of the feeding period, ten live honey bees were sampled from each cage and individually placed in 15 mL tubes. These samples were preserved in a liquid nitrogen container at −80 °C until further analysis.

To assess digestibility, protein concentrations in the hindgut of sampled honey bees were compared to concentrations in each diet [33]. Three iced honey bees were randomly chosen from each replication of sampled individuals, and the head and hindgut of each honey bee were extracted and weighted. Each diet was weighed at 200 mg for protein concentration measurements. Subsequently, iced 0.25 M Tris-HCl buffer at pH 7.5 was used for sample preparation. The honey bee tissues or diet samples were then homogenized in this buffer using a sterile disposable homogenizer included in the Ultra Grinder B kit (Taeshin Bioscience Co., Ltd., Seoul, Republic of Korea), along with 100 µL of buffer. The resulting mixture was diluted to a 20% solution with the same buffer, followed by centrifugation at 18,928× *g* for 30 s. The supernatant obtained after centrifugation was used to measure the protein concentration. The quantitative measurement of water-soluble protein was performed using the Pierce BCA Protein Assay Kit (Thermo Scientific, Rockford, IL, USA) [34]. The water-soluble protein content was analyzed following the method with minor modifications, and the absorbance at 562 nm was measured on a SynergyTM HTX Multi-Mode Reader (BioTeK, Santa Clara, CA, USA) [35]. The water-soluble protein content was calculated as µg/mL in the tissues. The approximate digestibility (%) was calculated using the equation: [(Protein concentration of diet − Protein concentration in hindgut)/Protein concentration of diet] × 100 [33,36].

### 2.5. Free Amino Acid Analysis

Samples (2 g) were subjected to extraction using a buffer solution containing 0.1 M perchloric acid and 0.1% meth-phosphoric acid in distilled water, followed by sonication for 1 h and continuous shaking for an additional hour at room temperature. The free amino acid contents of the diets were analyzed using an HPLC system equipped with an Inno C18 analytical column and employing gradient elution at Seoul National University. The analysis was conducted by the Official Methods of Analysis of the Association of Official Analytical Chemists (AOAC) and the Approved Methods of the American Association of Cereal Chemists (AACC), specifically by the AOAC method 994.12 [37]. Further details can be found in our previous paper [38].

### 2.6. RNA Extractions and Quantitative PCR (qPCR)

A representative fraction of the colony was sampled on 29 March (*n* = 3 colonies per diet treatment), with 20 honey bees per colony to obtain *vg* expression levels. A representative fraction of the cage was sampled on 17 May (*n* = 3 cages per diet treatment), with 10 honey bees per cage to obtain *vg* gene expression levels. All samples were vacuum-sealed into 15 mL conical tubes, which were promptly frozen on dry ice and kept at −80 °C for further analysis. From the honey bee samples collected from the field and cage experiments, nine honey bee abdomens each were dissected on dry ice. Total RNA was isolated from the pools of nine honey bee abdomens in each diet group using a Qiagen RNeasy Mini Kit (#74104; Qiagen, Valencia, CA, USA). The RNA concentration and purity were determined by measuring OD260/OD280 values within the range of 1.8 to 2.0.

The gene expression level of *vg* was measured by quantitative PCR (qPCR) using a cDNA template generated from total RNA. Using 1 μg of total RNA, cDNA was synthesized with oligo-dT with Invitrogen Superscript III enzyme (Grand Island, NY, USA). StepOne Plus (Applied Biosystems, Foster City, CA, USA) using SYBR green qRT-PCR Master Mix (Fermentas, Burlington, ON, Canada) was used under the following conditions: 95 °C for 5 min; 40 cycles of 95 °C for 30 s; 60 °C for 30 s; and 72 °C for 30 s. The primers for qRT-PCR are described in Table 2. The results were normalized to a validated control gene, β-actin, using the 2^(−ΔΔC(t))^ method [39]. All biological replicates were conducted in technical triplicate.

### 2.7. Statistical Analyses

The statistical analysis was performed using Microsoft Excel and XLSTAT software (Addinsoft Pearson Edition 2014, Addinsoft, Paris, France). Canonical discriminant analysis was applied to differentiate pollen substitute diets using the dataset comprising field and cage experiment parameters [40,41]. Principal Component Analysis (PCA) plots were generated from the field and cage experiment datasets, along with amino acid contents, to confirm the clustering of canonical discriminant analysis. Pairwise correlation analysis was conducted using the Pearson method [42] to identify significant relationships between traits (*p* < 0.05), with interpretation according to the guidelines provided by [43], categorizing correlations as very high positive (negative) correlation (±0.90 to 1.00), high positive (negative) correlation (±0.70 to 0.90), moderately positive (negative) correlation (±0.50 to 0.70), low positive (negative) correlation (±0.30 to 0.50), and negligible correlation (±0.00 to 0.30) [42]. Descriptive statistics were utilized to compute mean and standard deviation (SD) values, which were then visualized using the Heatmap module. The data were analyzed using SPSS version 25 (IBM), and Duncan’s multiple range tests were employed to detect significant differences between means at a significant level of *p* < 0.05. Graphs were created using GraphPad Prism software (version 7.03, Inc., San Diego, CA, USA).

## 3. Results

### 3.1. Colony Population

**Figure 2:**
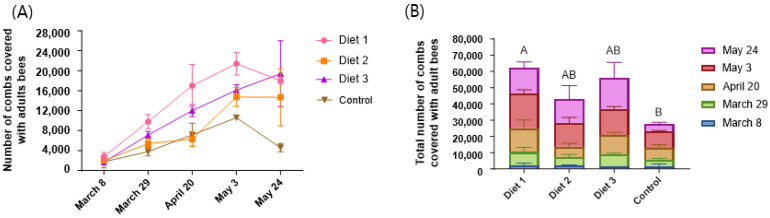
The number of combs covered with adult honey bees depends on the different diets. (**A**) The number of combs covered with adult honey bees according to feeding different kinds of diets on different days. (**B**) The total number of combs covered with adult bees during the experiment. The means followed by different letters are significantly different according to Duncan’s multiple range comparisons (DMRTs) (mean ± SD, *n* = 3) (*p* < 0.05).

The population size generally increased after feeding diets during the experiment periods (Figure 2A). However, the number of honey bees fed Diet 1 on 24 May (16,067 ± 3372 bees) was lower than that on 3 May (21,400 ± 2263 bees). Similarly, the number of honey bees fed the Control on 24 May (4600 ± 849 bees), which was similar to the count on 29 March (3800 ± 849 bees), was lower than that on 3 May (10,600 ± 283 bees). Consequently, we analyzed the total number of colony populations according to the diets. The results show statistically significant differences between the diets (*p* < 0.05) (Figure 2B). Diet 1 (62,517 ± 424 bees) showed the highest significant total number of honey bees, followed by Diet 3 (56,300 ± 11,597 bees), Diet 2 (43,113 ± 15,938 bees), and the Control (27,925 ± 3854 bees). Our development diets, Diet 1, Diet 2, and Diet 3, were significantly higher than the Control in terms of colony population.

### 3.2. Capped Brood Area

**Figure 3:**
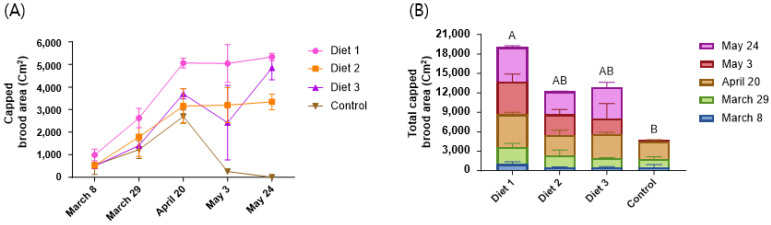
The capped brood area depends on the different diets. (**A**) Capped brood areas according to feeding different kinds of diets depend on the different times. (**B**) Total capped brood area according to feeding different kinds of diets during experiment season. The means followed by different letters are significantly different according to Duncan’s multiple range comparisons (DMRTs) (mean ± SD, *n* = 3) (*p* < 0.05).

The capped brood area generally increased during the experiment period in all our developed diet treatment groups (Figure 3A). The capped brood area of the colony-fed Diet 3 was lower on 3 May (2424 ± 2345 cm^2^) than on 20 April (3698 ± 321 cm^2^), but it increased on 24 May (4847 ± 749 cm^2^). The capped brood area of the colony-fed Control only exhibited brood area until 3 May during the experiment period. It increased until 20 April (2695 ± 286 cm^2^) but decreased on 3 May (255 ± 28 cm^2^). The total capped brood area exhibited statistically significant differences between the diets (*p* < 0.05) (Figure 3B). Diet 1 (19,054 ± 1346 cm^2^) demonstrated the highest total capped brood area, significantly surpassing Diet 3 (12,877 ± 2576 cm^2^), Diet 2 (12,249 ± 5005 cm^2^), and the Control (4704 ± 1060 cm^2^). Our developmental diets, Diet 1, Diet 2, and Diet 3, showed significantly larger total capped brood areas than the Control.

### 3.3. Consumption and Preference

**Figure 4:**
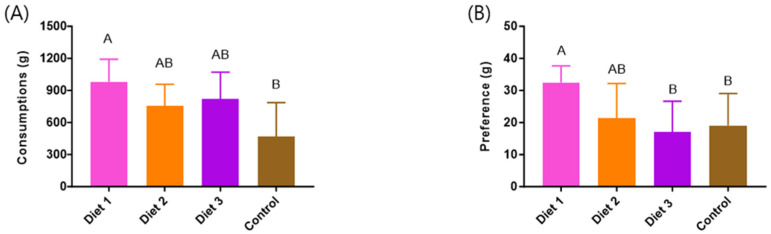
The consumption and preference of different types of diets in early spring (g/colony). (**A**) Consumption. (**B**) Preference. The means followed by different letters are significantly different according to Duncan’s multiple range comparisons (DMRTs) (*p* < 0.05) (mean ± SD, *n* = 3).

Significant differences were observed in the consumption of various diets (*p* < 0.05). Honey bees consumed significantly greater amounts of Diet 1 (980 ± 212 g), followed by Diet 3 (820 ± 251 g), Diet 2 (753 ± 205 g), and the Control (467 ± 320 g) (Figure 4A). Our developed diets, Diet 1, Diet 2, and Diet 3, showed significantly higher consumption than the Control. Regarding preference, significant differences were shown among the various diets (*p* < 0.05). Diet 1 (33 ± 5 g) showed the highest consumption among the diets, followed by Diet 2 (21 ± 11 g), the Control (19 ± 10 g), and Diet 3 (17 ± 10 g) (Figure 4B). Among our developed diets, Diet 1 and Diet 2 showed significantly higher preference than the Control.

### 3.4. Colony Weight

**Figure 5:**
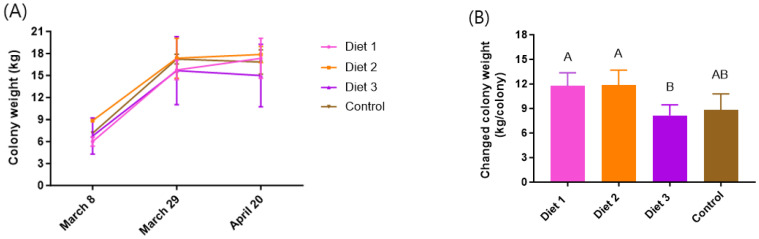
The colony weight depends on the different diets. (**A**) Colony weight according to different times depends on different diets. (**B**) Increased colony weight depends on the different diets from 8 March to 20 April. The means followed by different letters are significantly different according to Duncan’s multiple range comparisons (DMRTs) (*p* < 0.05) (mean ± SD, *n* = 3).

The colony weight increased during the experiment period in all treatment groups from 8 March to 20 April (Figure 5A). Total colony weight showed statistically significant differences between diets (*p* < 0.05) (Figure 5B). Colonies fed Diet 1 (12 ± 2 kg) and Diet 2 (12 ± 2 kg) exhibited statistically significant increases, ranking highest among the diets. The colonies fed Diet 3 (8 ± 1 kg) showed the least change in colony weight among the diets, with statistically significant differences observed, and this weight was lower compared to colonies fed the Control (9 ± 2 kg). Among our development diets, the colony-fed Diet 1 and Diet 2 were significantly heavier than the Control in terms of altered colony weight.

### 3.5. Honey Production

**Figure 6:**
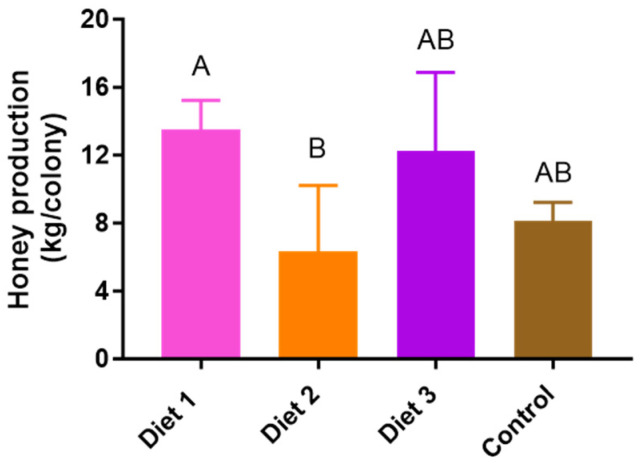
Honey production varies depending on the different diets during the blooming season of Acacia flowers from 3 May to 24 May. The means followed by different letters are significantly different according to Duncan’s multiple range comparisons (DMRTs) (*p* < 0.05) (mean ± SD, *n* = 3).

Significant differences were observed in the weight of honey production depending on the diets (*p* < 0.05) (Figure 6). Diet 1 (14 ± 2 kg/colony) exhibited the highest honey production weights, followed by Diet 3 (12 ± 5 kg/colony), the Control (8 ± 1 kg/colony), and Diet 2 (6 ± 3 kg/colony). Colonies fed Diet 1 had significantly higher honey production than those fed the Control.

### 3.6. Hygienic Behavior

**Figure 7:**
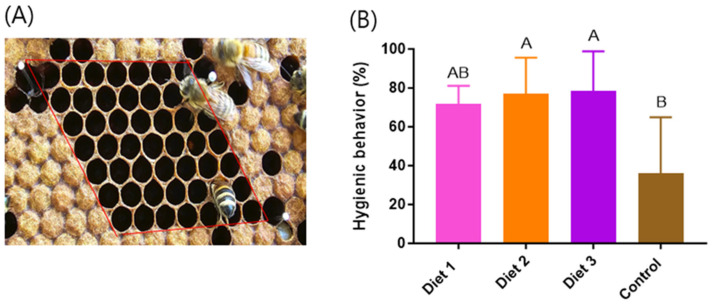
The hygienic behavior ability depends on different kinds of diets. (**A**) Here is a photo showing one hundred percent hygiene behavior ability taken 24 h after killing 49 cells. Inside the red lines indicates cleaned cells. (**B**) The results of hygienic behavior were assessed 24 h later to evaluate its effectiveness. The means followed by different letters are significantly different according to Duncan’s multiple range comparisons (DMRTs) (*p* < 0.05) (mean ± SD, *n* = 3).

The results show one hundred percent hygienic behavior ability due to cleaning 49 cells from 49 killing cells (Figure 7A). There were significant differences in the hygienic behavior ability percentage depending on the diets (*p* < 0.05) (Figure 7B). Diet 3 (78 ± 21%) exhibited the highest hygienic behavior percentage, followed by Diet 2 (77 ± 19%), Diet 1 (72 ± 10%), and the Control (36 ± 29%). Diet 2 and Diet 3 were the highest-level groups with statistical significance in terms of hygienic behavior percentage. All of our developed diets presented a higher-level group in hygienic behavior ability percentage with statistical significance compared to colonies fed the Control.

### 3.7. Dried Head and Thorax Weights

**Figure 8:**
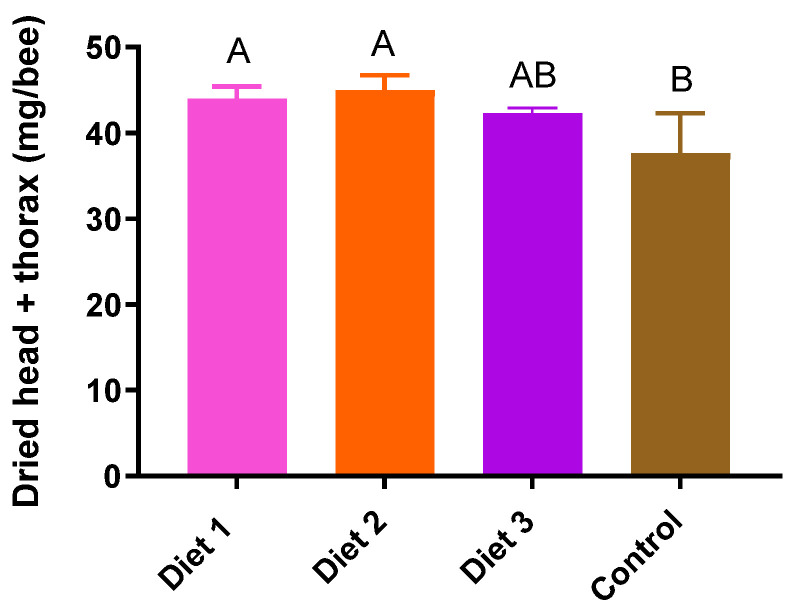
Weight of dried head and thorax of different diet types in early spring (8 March). The means followed by different letters are significantly different according to Duncan’s multiple range comparisons (DMRTs) (*p* < 0.05) (mean ± SD, *n* = 3).

There were significant differences in the dried head and thorax weight according to the diets (*p* < 0.05) (Figure 8). Diet 2 (45 ± 2 mg/bee) exhibited the highest dry head and thorax weights, followed by Diet 1 (44 ± 1 mg/bee), Diet 3 (42 ± 1 mg/bee), and the Control (38 ± 5 mg/bee). Diet 1 and Diet 2 were significantly heavier in terms of dried head and thorax weights compared to the other diets. All of our developed diets showed significantly higher dried head and thorax weights compared to colonies fed the Control.

### 3.8. Vitellogenin (vg) Gene Expression Level

**Figure 9:**
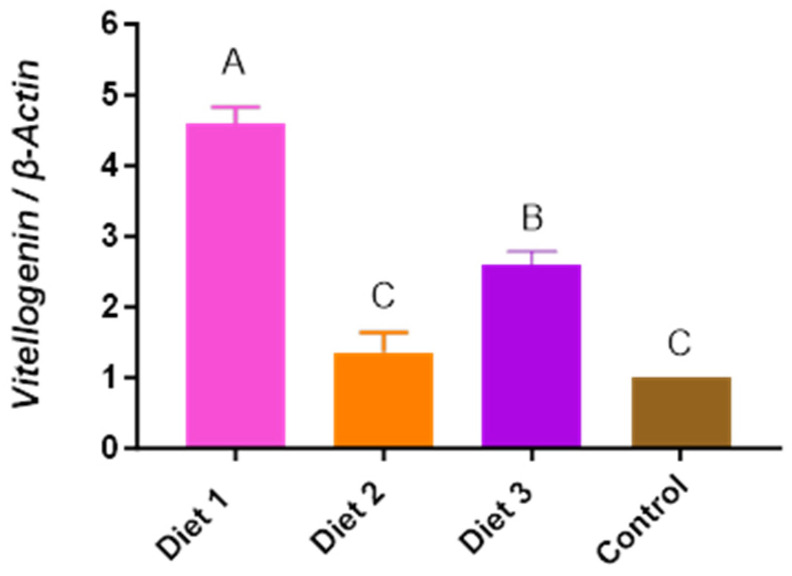
The response of honey bee vitellogenin gene expression to different diets in early spring (29 March). The means followed by different letters are significantly different according to Duncan’s multiple range comparisons (DMRTs) (*p* < 0.05) (mean ± SD, *n* = 3).

The *vg* gene expression level exhibited significant differences depending on the diets (*p* < 0.05) (Figure 9). Diet 1 (4.58 ± 0.25) exhibited the highest *vg* expression levels, followed by Diet 3 (2.61 ± 0.18), Diet 2 (1.36 ± 0.28), and the Control (1). Honey bees fed Diet 1 showed the highest *vg* expression level among all groups, with statistical significance. Both honey bees fed Diet 1 and Diet 3 exhibited a significantly higher level of *vg* expression compared to colonies fed the Control.

### 3.9. Cage Experiments

**Figure 10:**
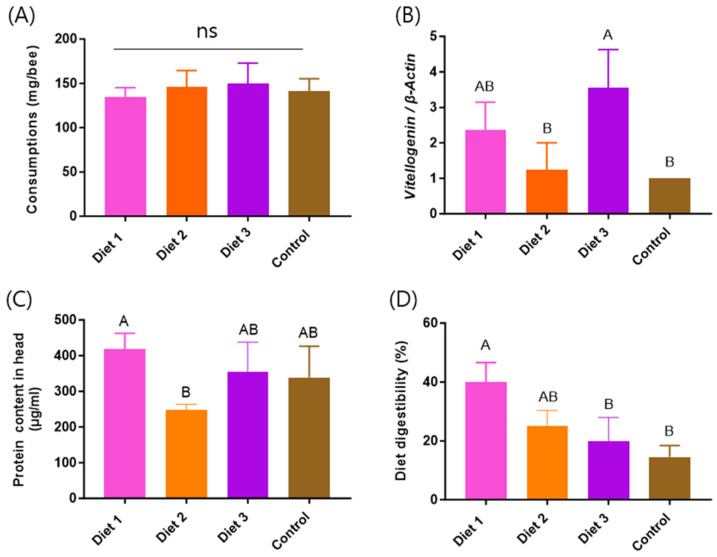
The response of honey bees to different pollen substitute diets in cage experiments during the spring (17 May). (**A**) Consumption. (**B**) Vitellogenin gene expression level. (**C**) Protein content in the head. (**D**) Diet digestibility (%). The means followed by different letters are significantly different according to Duncan’s multiple range comparisons (DMRTs) (*p* < 0.05) (mean ± SD, *n* = 3).

In the cage experiments, consumption, *vg* gene expression level, protein content in the head, and diet digestibility were assessed depending on the pollen substitute diets. Among these indicators, *vg* gene expression level, protein content in the head, and diet digestibility showed statistically significant differences among the diets (*p* < 0.05) (Figure 10). Regarding consumption, no significant difference was observed among the diets (Figure 10A). Concerning the *vg* expression level, the cage group fed Diet 3 (3.55 ± 1.08) exhibited the highest levels, followed by Diet 1 (2.36 ± 0.79), Diet 2 (1.23 ± 0.77), and the Control (1). The cage group fed Diet 3 showed significantly higher *vg* expression levels compared to the other diets, and both Diet 1 and Diet 3 showed significantly higher levels compared to the cage group fed the Control. As for protein content in the head, the cage group fed Diet 1 (418.44 ± 44.03 μg/mL) exhibited the highest levels, followed by Diet 3 (355.76 ± 81.72 μg/mL), the Control (338.58 ± 87.50 μg/mL), and Diet 2 (246.63 ± 16.77 μg/mL). The cage group fed Diet 1 demonstrated the highest levels of protein content in the head, with statistical significance. Additionally, both Diet 3 and the Control showed significantly higher levels compared to cages fed Diet 2.

In terms of diet digestibility, the cage group fed Diet 1 (39.99 ± 6.57%) exhibited the highest level, followed by Diet 2 (25.02 ± 5.32%), Diet 3 (19.76 ± 8.18%), and the Control (14.49 ± 3.90%). Diet 1 demonstrated the highest levels of diet digestibility, with statistical significance. Additionally, both Diet 2 and Diet 3 showed significantly higher levels of diet digestibility compared to colonies fed the Control.

### 3.10. Amino Acid Content Analysis

**Figure 11:**
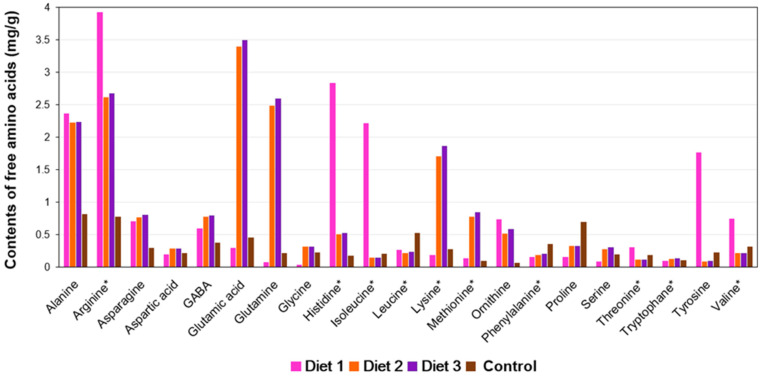
Contents of free amino acids in different pollen substitute diets (mg/g). * Indicates essential amino acids for honey bees [19]. Ornithine is a non-proteinogenic amino acid, and GABA is not an amino acid but the amine of butyric acid and a neurotransmitter in function.

The content of the free amino acids in the pollen substitute diets was analyzed, revealing the presence of twenty-one different amino acids (Figure 11). Diet 3 (18.69 mg/g) exhibited the highest total free amino acid content, followed by Diet 2 (17.95 mg/g), Diet 1 (17.76 mg/g), and the Control (6.67 mg/g). Notably, Diet 1 showed the highest levels of essential amino acids, including arginine (3.92 mg/g), histidine (2.83 mg/g), isoleucine (2.21 mg/g), and valine (0.74 mg/g), as well as the non-essential amino acid tyrosine (1.76 mg/g), among the treated diets. Diet 2 and Diet 3 were found to have the highest levels of essential amino acids, including lysine (1.70 mg/g and 1.86 mg/g) and methionine (0.77 mg/g and 0.84 mg/g), as well as non-essential amino acids, including glutamic acid (3.39 mg/g and 3.49 mg/g) and glutamine (2.48 mg/g and 2.59 mg/g), among the tested diets, respectively. The Control showed the highest levels of essential amino acids, including leucine (0.52 mg/g) and phenylalanine (0.35 mg/g), as well as non-essential amino acids, including proline (0.69 mg/g), among the tested diets.

### 3.11. Discrimination of Diet Effects by Canonical Discriminant Analysis and Principle Component Analysis

**Figure 12:**
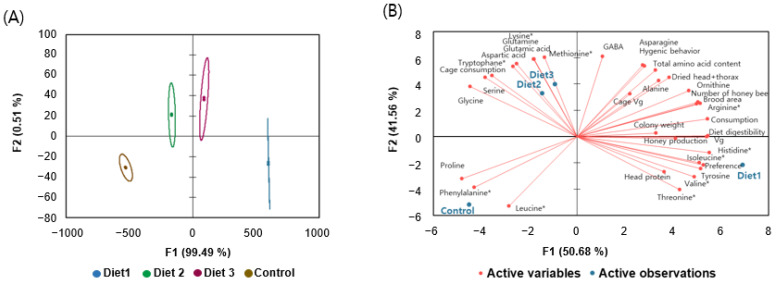
(**A**) Canonical discriminant analysis of field and cage experiments depends on the different pollen substitute diets. (**B**) Principle component 2D biplot graphs for various traits of pollen substitute diets and effects on honey bees. * Indicates essential amino acids for honey bees [19].

Each diet was differentiated through comparisons of field and cage experiment results via canonical discriminant analysis (Figure 12A). The Wilks’ lambda test was utilized to evaluate the significance of the discriminant model in our analysis. The observed value of Wilks’ lambda was determined to be 0.00, indicating a highly significant difference among diets based on the predictor variables. This finding was further supported by the F-statistic, which had an observed value of 269.07, far exceeding the critical value of 6.86 at a significance level of alpha = 0.05. The degrees of freedom for the F-statistic were 24 and 3.50 for the numerator and denominator, respectively. The *p*-value associated with the F-statistic was less than 0.0001 ***, indicating an extremely significant result. This provides strong evidence against the null hypothesis of no difference among diets. Consequently, we reject the null hypothesis and conclude that there are significant differences in the predictor variables across diets. The discriminatory power of the F1 function was high (eigenvalue of 253,470.01), explaining 99.49% of the total variation, with the second function (F2) contributing 0.51% of the remaining variation. Through these results, we confirmed that Diet 1 was distinct from the other diets.

Principle component analysis (PCA) was conducted using two main principle factors (F1 and F2) for every indicator, including field experiments, cage experiments, and free amino acid contents of each diet (Figure 12B). The PCA biplot for all indicators accounted for 92.24% of the total variability, revealing that histidine, diet digestibility, consumption, *vg* gene expression levels, and isoleucine were the most discriminating parameters of Diet 1 from the other tested diets.

### 3.12. Heat Map Depicting the Effect of Pollen Substitute Diets

**Figure 13:**
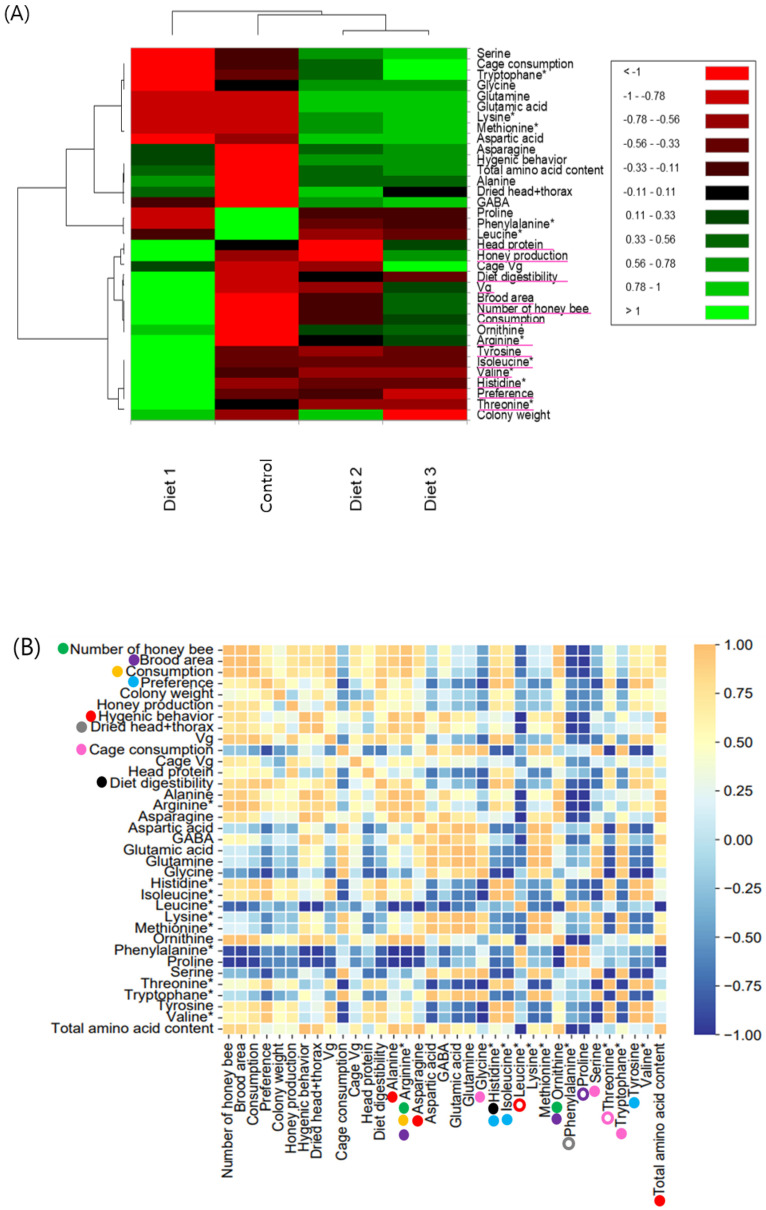
Visualization depicting the effects of pollen substitute diets. (**A**) Visualization of the impact of pollen substitute diets using a heat map. The tested indicators are listed on the right side, and the pink line under the indicators helps to distinguish Diet 1 from the other diets. (**B**) Correlation matrix illustrating the effects of different diets. The filled circles of the same color next to the indicators represent statistically significant positive correlations, while the outlined circles of the same color denote statistically significant negative correlations (*p* < 0.05). * Indicates essential amino acids for honey bees [19].

Following heat map analysis, each diet was distinguished based on all tested indicators. Diet 1 clustered separately from the other diets across all indicators (Figure 13A). Notably, among all indicators, the following traits highly contributed to the separation of Diet 1 from the other diets: head protein, honey production, diet digestibility, *vg* gene expression level, brood area, number of honey bees, consumption, arginine, tyrosine, isoleucine, valine, histidine, preference, and threonine.

A correlation matrix was constructed using all indicators of pollen substitute diets (Figure 13B). In the field and cage experiments, significantly positive correlations were observed between the number of honey bees, brood area, and consumption (*p* < 0.05). Additionally, a strong positive correlation (*r* = 0.996) was found between *vg* gene expression level and consumption.

In comparison, amino acid contents and all indicators of experiments, including non-essential amino acids alanine and asparagine, and total amino acid content, were significantly positively correlated with hygienic behavior (*r* = 0.973, *r* = 0.999, and *r* = 0.995, respectively), whereas the essential amino acid leucine showed a negative correlation (*r* = −0.996). Furthermore, the essential amino acid arginine exhibited significantly positive correlations with the number of honey bees, brood area, and consumption (*r* = 0.960, *r* = 0.983, and *r* = 0.965, respectively). Histidine, another essential amino acid, was significantly positively correlated with preference (*r* = 0.958) and diet digestibility (*r* = 0.955), while isoleucine, also an essential amino acid, and the non-essential amino acid tyrosine showed significant positive correlations with preference (*r* = 0.966 and *r* = 0.961, respectively). Ornithine, a non-essential amino acid, exhibited a significant positive correlation with the number of honey bees (r = 0.964), brood area (*r* = 0.982), the non-essential amino acid alanine (*r* = 0.971), and the essential amino acid arginine (*r* = 0.982). Phenylalanine, an essential amino acid, showed a significantly negative correlation with dried head and thorax weight (*r* = −0.956), while proline, a non-essential amino acid, exhibited a significantly negative correlation with brood area (*r* = −0.974). Lastly, serine, a non-essential amino acid, and tryptophan, an essential amino acid, were significantly positively correlated with cage consumption (*r* = 0.994 and *r* = 0.981, respectively), whereas threonine, an essential amino acid, showed a significantly negative correlation with cage consumption (*r* = −0.965).

## 4. Discussion

It is essential to validate the effects of pollen substitute diets on honey bees through both field and cage experiments because results from laboratory studies may not necessarily reflect real field conditions [15]. The reliability of results increases when findings from laboratory and field studies are consistent. However, divergences between the two experiments can provide insights into environmental factors that impact the efficacy of pollen substitutes. In this study, the results provide valuable insights into enhancing honey bee health through field and cage experiments. The analysis also compared amino acid content across different pollen substitute diets due to essential nutrient sources [39].

Overall, based on the results of both field and cage experiments, Diet 1 demonstrated superior performance. In field experiments, honey bees fed Diet 1 demonstrated generally favorable performance, including the number of honey bees, brood area, consumption, preference, colony weight, honey production, dried head and thorax, and *vg* gene expression levels. Similarly, in cage experiments, honey bees fed Diet 1 exhibited a high level of protein content in the head and diet digestibility. The statistical analysis, employing canonical discriminant analysis, effectively distinguished between diets based on the predictor variables, supported by the low Wilks’ lambda value and significant F-statistic. The results of the Wilks’ lambda test indicate that the discriminant model effectively differentiated between diets based on the predictor variables, thus validating the discriminant analysis. To further elucidate the distribution of the canonical discriminant analysis results, PCA was conducted. Utilizing PCA with two main principal factors (F1 and F2) for each indicator provided additional insights into the dataset’s variability. The results highlighted Diet 1 as distinct from the Control, with Diet 2 and Diet 3 occupying intermediate positions between Diet 1 and the Control. Among all parameters, histidine, diet digestibility, consumption, *vg* gene expression levels, and isoleucine were identified as the most discriminating factors through PCA, serving as markers that distinguish Diet 1 from the other tested diets.

Furthermore, the correlation matrix reveals that among the investigated amino acids, arginine exhibited the most significant correlations with a wide range of parameters, including the number of honey bees, brood area, and consumption. Additionally, histidine exhibited a highly positive correlation with preference and diet digestibility. Specifically, histidine and diet digestibility emerged as the top discriminating parameters distinguishing Diet 1 from the other diets based on PCA. In another study, it was identified that honey bees preferred proline-containing artificial nectars above alanine and serine, and alanine above serine [22]. In other studies, it was found that histidine contents were notably low in maize pollen, resulting in higher mortality among honey bees fed maize pollen compared to those fed mixed pollen [44]. Further research is needed to elucidate the effect of histidine on honey bee physiology and various aspects.

Arginine and ornithine exhibited significant positive correlations with the number of honey bees and brood area, metrics commonly used to assess colony strength, which is the primary goal of feeding pollen substitute diets [15]. These two amino acids were found to be the highest in Diet 1 compared to the other diets, potentially contributing to the high level of measures associated with colony development. In another study, dietary supplementation with arginine was found to have specific effects on the pupation rate, emergence rate, antioxidant capacity, and immunity of honey bee larvae [45,46]. However, further research is needed to understand the specific impact of these amino acids on honey bee health and development.

In a separate study, while β-alanine, GABA, citrulline, ornithine, and taurine did not affect nectar palatability, β-alanine and GABA were observed to improve learning and memory functions in honey bees [47]. Additionally, β-alanine was found to improve the survival rate of newly emerged honey bees [48]. In the present study, alanine, asparagine, and the total amino acid content exhibited a significant positive correlation with hygienic behavior, while leucine exhibited a significant negative correlation with hygienic behavior.

Honey bees fed Diet 1 generally outperformed bees fed on the other diets, however, hygienic behavior was slightly lower compared to Diet 2 and Diet 3. In the amino acid contents of Diet 1, asparagine and the total amino acid content were lower than in Diet 2 and Diet 3. Further research is needed to clarify the effect of asparagine and total amino acid content on hygienic behavior for more detailed confirmation. Hygienic behavior in honey bees is a multifaceted trait influenced by multiple genes, acting as a built-in defense mechanism against bacterial and fungal infections in brood cells, as well as infestations by *Varroa destructor* [49]. Thymol has demonstrated potential in improving hygienic behavior, achieving a maximal removal rate of 88% [50]. It appears that incorporating thymol into the developed pollen substitute diets could potentially create new diets in the future that enhance hygienic behavior.

In both field and cage experiments, consumption and *vg* expression levels serve as commonly evaluated indicators. In the field experiment, honey bees fed Diet 1 displayed the highest consumption rate, while no significant differences were observed among the diets in the cage experiment. In the cage experiments, only honey bees of the same age are used, whereas different ages of honey bees live together in hives. This suggests that dietary consumption may vary depending on environmental conditions and colony dynamics. The consumption rate is a vital metric for gauging diet attractiveness and nutritional value [15]. However, in the cage experiments, honey bees fed Diet 1 exhibited higher protein content in the head. In another study, nurse bees digested approximately 35% of the water-soluble protein in two artificial diets [33]. In this study, honey bees displayed around 40% digestibility for water-soluble protein. Digestibility is linked to nutrient absorption levels, influencing nutrient uptake by the body’s cells post-digestion. In honey bees, the development of hypopharyngeal glands, responsible for royal jelly secretion crucial for larval nutrition, correlates with protein content in the head [51,52]. Worker honey bee larvae are typically fed royal jelly initially, transitioning to a diet including honey and pollen [53]. It can be inferred that the protein content in the head correlates with the development of these glands, thereby influencing colony development [54]. It can be speculated that Diet 1 may contribute to the development of hypopharyngeal glands, potentially impacting colony development.

Regarding *vg* gene expression levels, honey bees fed Diet 1 exhibited the highest levels in the field experiment, whereas Diet 3 led to the highest expression in cage conditions. *vg*, primarily involved in egg yolk production, influences various physiological processes, including nursing behavior and social organization longevity in honey bees [55]. Discrepancies in *vg* expression levels between field and cage experiments may result from environmental disparities, particularly the presence of brood care duties for nurse bees in the colony environment [39]. These environmental differences likely influence the behavioral and physiological responses of honey bees, leading to variations in *vg* expression levels observed between field and cage conditions.

The honey bee serves as a crucial model organism for studying social behavior, particularly in locating food sources, a process intricately linked to gustatory sensing areas [56]. With 12 gustatory receptor (*Gr*) genes identified in honey bees, *AmGr10* stands out as a key player functioning as an amino acid taste–sensing receptor, distinct from its response to sweet and bitter compounds [39]. Its expression extends across various structures on the head, body, and legs, with enrichment observed in internal organs, such as the fat body, brain, and hypopharyngeal gland [39]. These internal domains likely play a role in monitoring amino acid concentration and intestinal absorption or metabolism. Further exploration is necessary to elucidate the mechanism underlying the interaction between *AmGr10* and nutrient absorption, which could significantly contribute to understanding and promoting honey bee health.

In our prior study, Diet 2, incorporating apple juice as an additive, exhibited the best performance among our developed pollen substitute diets, followed by Diet 3 with soytide as an additive [26]. However, our current study demonstrates that Diet 1, which combines apple juice, soytide, and *Chlorella*—additives previously proven effective—outperformed Diet 2 and Diet 3. This suggests an enhanced formulation for pollen substitute diets such as Diet 1. It is interesting that our diet components are similar, but Diet 1 and Diet 3 included probiotics such as soytide powder instead of defatted soybean powder. Additionally, Diet 1 had *Chlorella* powder added specifically. However, there were significant differences in some amino acids, including histidine, isoleucine, tyrosine, valine, and arginine. We speculate that these differences are due to the probiotics because they can affect the production of amino acids and vitamins [57]. In our three pollen substitute diets, we included brewer’s yeast and soybean-related powder. If these diets are continued to be fed to the colony during the blossom season, brewer’s yeast cells and soybean particles can be found in the honey through microscopic pollen analysis. Consequently, the honey would no longer comply with legislation (Directive 2001/110 and Codex Alimentarius definition of honey). Therefore, these pollen substitute diets should be utilized in colonies during periods of pollen scarcity in early spring and particularly during weakened conditions (before the blooming season and after), not during the blooming season. Overall, our findings suggest that the developed pollen substitute diet has the potential to significantly improve colony health and development.

## 5. Conclusions

Our study highlights the importance of thoroughly evaluating pollen substitute diets through both field and cage experiments. While these diets contain various nutrients, proteins, as a critical component, have a significant impact on body development and physiology, essential for brood rearing and colony development in honey bees. Among the tested diets, Diet 1 demonstrated superior performance across various parameters related to honey bee health and development. Specific amino acids displayed a significant correlation with honey bee health-related parameters. Specifically, in this study, all results were compared based on amino acid contents. Due to limited research on the effects of individual amino acids on honey bees, further detailed studies on their physiological and behavioral responses, especially under various stress factors, are warranted. Incorporating such findings could enhance our understanding of honey bee health and contribute to the development of more effective pollen substitute diets in the future.

## 6. Patents

We have patents related to the diets in the present study titled “Bee Feed Composition”, granted on 12 December 2022 (No. 10-2022-0166677).

## Figures and Tables

**Table 1:** Formulation of pollen substitute diets using consistent ingredients (%).

Ingredients	Diet 1	Diet 2	Diet 3
Brewer’s yeast	39.69	39.69	39.69
Egg yolk powder	2.21	2.21	2.21
Defatted soybean powder	-	2.21	-
Sugar	35.36	35.36	35.36
Boiled water	7.16	7.16	5.16
Canola oil	1.01	1.01	1.01
Cellulose	0.88	0.88	0.88
Wheat bran powder	0.88	0.88	0.88
Multiple vitamins	0.44	0.44	0.44
L-methionine	0.10	0.10	0.10
L-lysine	0.24	0.24	0.24
Citric acid	1.85	1.85	1.85
Tangerine juice	4.00	4.00	10.00
Soytide powder	2.21	-	2.21
Apple juice	4.00	4.00	-
*Chlorella* powder	0.08	-	-

**Table 2:** PCR primer information.

Gene		Primer Sequence (5′-3′)	Size (bp)
*vg*	F R	GTTGGAGAGCAACATGCAGA TCGATCCATTCCTTGATGGT	150
*ß-actin*	F R	AGGAATGGAAGCTTGCGGTA AATTTTCATGGTGGATGGTGC	181

## Data Availability

The data that are presented in this study are available in the article.
